# Systematic review and meta-analysis of candidate gene association studies of benign prostate hyperplasia

**DOI:** 10.1186/s13643-022-01914-7

**Published:** 2022-04-05

**Authors:** Lin Lin, Pugui Li, Xin Liu, Xiuyuan Xie, Liping Liu, Anjani Kumar Singh, Himanshu Narayan Singh

**Affiliations:** 1 Department of Urology, Hospital of Traditional Chinese and Western Medicine of Taizhou, Taizhou, 317502 Zhejiang China; 2 Department of Urology, Tangdu Hospital,Air Force Military Medical University, Xi’an, Shaanxi 710032 China; 3 Department of Critical Care Medicine, People’s Hospital of Xinjiang Uygur Autonomous Region, Urumqi Municipality, 830000 Xinjiang China; 4 Department of Urology, The First Affiliated Hospital of Nanjing Medical University, Nanjing City, 210029 Jiangsu China; 5 Department of Outpatient, The Second Affiliated Hospital of Guangzhou Medical University, Guangzhou City, 510260 Guangdong China; 6 Department of Physics, Atma Ram Sanatan Dharma College, University of Delhi, New Delhi, India; 7 TAGC, Aix Marseille University, Marseille, France

**Keywords:** Benign prostate hyperplasia, Genetic polymorphism, Lower urinary tract symptoms, Systematic review, Meta-analysis

## Abstract

**Background:**

Benign prostate hyperplasia (BPH) is the most common urological problem in elderly males. Recent studies have reported polymorphism in various metabolic genes in BPH. However, their association with the susceptibility of BPH is still inconsistent. Here, we systematically reviewed and performed a meta-analysis of CYP17, VDR, and ACE genes to determine their precise association with the risk of BPH.

**Methods:**

A comprehensive literature search for published studies on candidate gene associations involving vitamin D receptor (VDR), angiotensin-converting enzyme (ACE), and CYP17 genes with the risk of BPH was done up to April 2020 in PubMed, Scopus, Cochrane Central Register of Controlled Trials (CENTRAL), and Google Scholar databases. Fixed/random effects models were used to estimate the odd’s ratio (OR) and 95% confidence intervals (CIs). Begg’s funnel plot was used to assess the potential for publication bias.

**Results:**

We found a total of 23 studies containing 3461 cases and 3833 controls for these gene polymorphisms. A significant association of ACE gene polymorphism was observed under the recessive (II vs. ID + DD) model for BPH susceptibility compared to control subjects (overall *OR* = 1.67, 95% *CI* = 1.03–2.73). Similar trends were observed for ACE gene polymorphism in Caucasian (*OR* = 6.18, 95% *CI* = 1.38–27.68) and Asian (*OR* = 1.42, 95% *CI* = 0.99–2.03) populations under study. No significant association was observed in VDR and CYP17 gene polymorphisms in any dominant or recessive models.

**Conclusion:**

Significant OR demonstrated the implication of ACE gene polymorphism in the proliferation of prostate tissue, which in turn is associated with BPH susceptibility. However, prospective studies at large scale and sample size are needed to confirm the current findings.

**Supplementary Information:**

The online version contains supplementary material available at 10.1186/s13643-022-01914-7.

## Introduction

Benign prostatic hyperplasia (BPH) is one of the most common diseases in lower urinary tract symptoms (LUTS), in middle-aged and elderly men. It is a non-malignant enlargement of the prostate which can cause urinary dysfunction and may affect the quality of life of patients [1]. Being a progressive disease, it results in more severe LUTS, making the life of patients more difficult and after the age of 80 years; various treatments are given to patients according to their symptoms. BPH is a multifactorial and complex disease. Roles of ageing, heritability, ethnicity, and family history have been demonstrated in BPH development [2]. Still, its aetiology remains unclear. However, recent literature demonstrates the role of gene polymorphisms, metabolic changes, and inflammation among ageing males in BPH [3].

Androgens are required for normal growth and development of the prostate gland and even reported in the maintenance of BPH [4]. The *CYP17* gene codes for the cytochrome P450c17α enzyme, which plays a crucial role in the synthesis of testosterone from its precursor cholesterol. The CYP17 gene, in its 5′-untranslated region, harbours a nucleotide substitution (rs743572) of ‘T’ (A1 allele) to ‘C’ (A2 allele) resulting in higher levels of androgens. This change in nucleotide results in a new Sp-1 site (CCACC box) at 34 bp upstream of the translation site and downstream of the transcription site, which in turn acts as an additional promoter elevating CYP17 transcription [5]. Several studies have investigated the association between CYP17 rs743572 polymorphism and BPH susceptibility, but still there is no clarity.

Another metabolic factor, angiotensin-converting enzyme (ACE), plays a significant role in “classical” renin–angiotensin–aldosterone system (RAAS) through which the proliferation of cellular elements in the prostate is regulated [6]. One of the well-known polymorphisms in the ACE gene is the insertion/deletion polymorphism, which is 287 bp long and results in three genotypes (II, ID, and DD). These genotypes have been shown to be associated with ACE activity and levels in plasma and tissues. The primary transcript of the gene is differentially spliced, and different versions of the mature mRNAs are translated to synthesize various isoforms of the enzyme; of them, one isoform is adequately expressed in the testis. This polymorphism of the ACE gene has been associated with an increased risk for many diseases [7, 8]. High levels of ACE are associated with BPH [9]. The hyperactivity of local tissue RAAS is considered involved in the pathophysiology of BPH [10]. Considering the suggested involvement of RAAS in BPH, the ACE insertion/deletion (I/D) polymorphism could have implications in the pathophysiology of BPH.

Vitamin D and its analogues possess antiproliferative and differentiation effects on prostate cells in both in vivo and in vitro [11, 12]. The antineoplastic actions of vitamin D appear to be mediated primarily through the vitamin D receptor (VDR), which is a member of the steroid/thyroid hormone receptor superfamily. Low vitamin D level is an independent risk factor for BPH [13]. More than 11 single nucleotide polymorphisms (SNPs) have been reported in VDR gene’s coding and promoter [14]. Several studies have been done investigating their role in the progression of the BPH [15–18]. Still, there is no consensus between VDR polymorphism and the risk of BPH.

Therefore, we performed the present meta-analysis based on the multivariate method to evaluate the possible role of CYP17, VDR, and ACE gene polymorphisms towards the risk of BPH.

## Methods

### Literature search

This systematic literature review was performed using the guidelines of the PRISMA statement (Preferred Reporting Items for Systematic Reviews and Meta-analyses) [19] and Cochrane Handbook for Systematic Reviews of Intervention. An electronic search of PubMed, Scopus, Cochrane Central Register of Controlled Trials (CENTRAL), and Google Scholar databases was performed for English language papers published up to 31 April 2020. The following key terms were used: ‘Candidate Association studies OR ‘cytochrome P450c17alpha’ OR ‘Angiotensin Converting Enzyme’, OR ‘Vitamin D Receptor’ OR ‘Gene Polymorphisms’ AND ‘Benign Prostate Hyperplasia’. Additionally, the reference list of retrieved studies, review articles, and previous meta-analyses were manually searched for collecting more relevant studies often missed while performing the electronic search. During the literature search, all candidate gene association studies involving VDR, ACE, and CYP17 polymorphisms on the risk of benign prostate hyperplasia compared to the control group were included, whereas duplicates, case reports, and case series were excluded.

#### Selection criteria

Case–control candidate gene association studies reporting genetic polymorphisms of VDR, ACE, and CYP17 polymorphisms with the risk of BPH were included. Two authors independently and in duplicate screened titles, abstracts, and full texts determined eligibility, abstracted data, and assessed the credibility of pooled associations. Meta-analyses were performed for genetic variants assessed in more than two studies.

### Data extraction

Two investigators independently extracted the data. The following data were extracted from each study: first author’s name, published year, ethnicity, country, number of cases and controls, mean age, genotyping method, source of control population, either hospital- or population-based, and frequency distribution of selected polymorphisms. Hardy–Weinberg equilibrium (HWE) was calculated for the allelic frequency distribution. Ethnicities were categorized as Asian and Caucasian populations.

### Quality assessment

The Newcastle–Ottawa Scale (NOS) [20] was used for assessing the quality of the included studies based on three components: selection, comparability, and ascertainment of outcome. Scores ranged from 01 to 09. Two authors independently assessed the quality of the included studies. Discrepancies over quality scores were resolved by discussion among all authors and a subsequent consensus was reached.

#### Risk of bias assessment

The risk of bias was assessed with Newland Ottawa Scale, and publication bias was assessed using Begg’s and Egger’s funnel plot analysis.

### Statistical analysis

Odds ratios (ORs) with 95% confidence intervals (CIs) were calculated to investigate the relationship between ACE, Cyp17, and ACE gene polymorphisms and the risk of BPH using fixed (Mantel–Haenszel method) [21] or random effects (Dersimonian and Laird method) models [22]. Heterogeneity between the studies was compared by using Cochran’s-*Q* statistic and *I*^2^ metric [23, 24]. The *I*^2^ metric was used to describe the degree of heterogeneity between the included studies, where 0–25% indicated no observed heterogeneity and larger values showed increasing heterogeneity, with 25–50% regarded as low, 50–75% as moderate, and 75–100% as high. Heterogeneity between studies was adjusted by subgroup analysis, HWE status, and meta-regression by quality score of the included studies.

One-way sensitivity analyses were performed to assess the stability of the results, namely, a single study in which the meta-analysis was deleted each time to reflect the influence of the individual dataset on the pooled OR. Funnel plots and Egger’s linear regression test were used to obtain the potential publication bias [25, 26]. The presence of selection bias in control participants was evaluated by calculating HWE, and genotypic frequencies of the control subjects were compared by using the chi-square test. Stratified analysis based on ethnicity (Asian versus Caucasian) was performed. To ensure the reliability and accuracy of the results, two investigators entered data into the software and reached a consensus. All statistical analyses were performed using STATA 13.0 software. *P* value <0.05 was considered statistically significant.

## Results

### Literature search

**Fig. 1:**
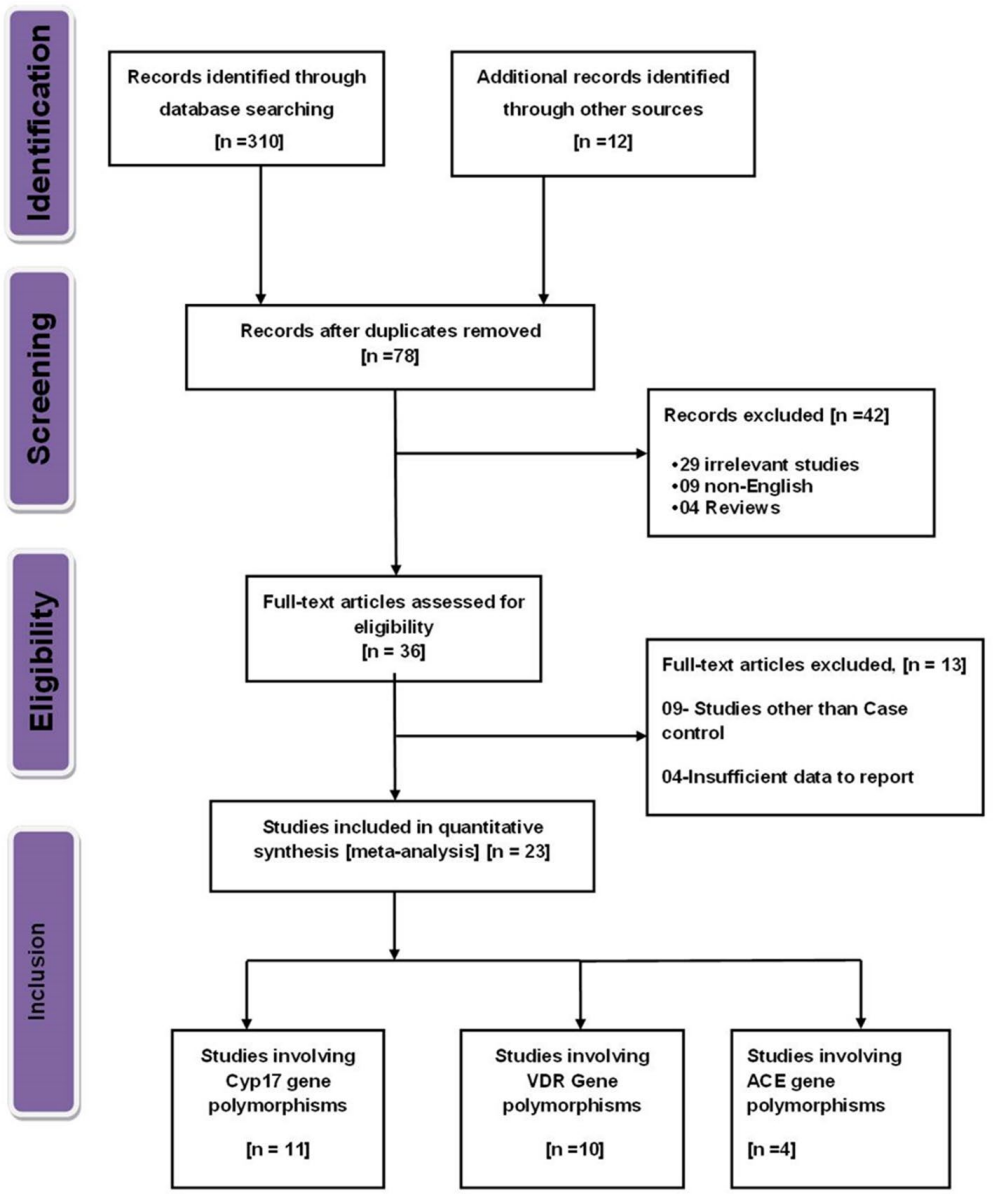
Flow diagram for the selection of studies and specific reasons for exclusion from the present meta-analysis

The initial search yielded 310 records from PubMed, Embase, Scopus, Web of Science Cochrane Central Register of Controlled Trials (CENTRAL), and Google Scholar databases. Of them, 281 were excluded after the review of the title/abstract, leaving 29 potential studies for full-text information review. Finally, 23 studies met the inclusion criteria and were included in this study (Fig. 1).

### Characteristics of eligible studies

The main characteristics of the included studies are presented in Table 1. The publication years of the studies included in our analysis ranged from 2000 to 2020. The sample size in each study ranged from 20 to 588. Total 23 case–control studies, 11 for CYP17 [2, 5, 16, 27–34], 10 for VDR [15–17, 34–40], and 4 studies for ACE I/D [41–44] polymorphisms were included in our meta-analysis (Table 1). Studies were carried out in two major ethnic populations; 13 studies were in Asian while 10 studies were in the Caucasian population. All studies in this meta-analysis had controls in HWE. The quality scores of all included studies were moderately high. Out of 23 studies, 21 studies were hospital-based, 02 studies were population-based, and 01 study had a mixed source of controls. Table 1 gives a summary of the characteristics and methodological quality of all included studies.

**Table 1:** Characteristic of included studies in a meta-analysis

S. no	Ref. no.	Country	Ethnicity	Total cases	Total control	Genes	Variant studied	Genotype method	Age case [mean ± SD]	Age control [mean ± SD]	HWE	Source of control	NOS quality score
Studies included for the CYP17 gene polymorphism													
1.	[2]	Iraq	Caucasian	50	50	CYP17	34 A1/A2	PCR-RFLP	45-80	45–60	YES	HB	5
2.	[5]	India	Asian	100	100	CYP17	34 A1/A2	PCR-RFLP	63.9 ± 8.9	64.1 ± 7.8	YES	NR	6
3.	[16]	Lebanon	Caucasian	68	79	CYP17	34 A1/A2	PCR-RFLP	65.98 ± 9.97	58.33 ± 10.14	YES	Mixed	7
4.	[27]	Japan	Asian	202	131	CYP17	34 A1/A2	PCR	70.5 ± 9.4	75.3 ± 7.3	YES	HB	6
5.	[28]	China	Asian	182	274	CYP17	34 A1/A2	PCR-RFLP	70 [53-94]	NA	YES	PB	7
6.	[29]	Turkey	Caucasian	80	73	CYP17	34 A1/A2	PCR	NA	NA	YES	NR	5
7.	[30]	Turkey	Caucasian	136	102	CYP17	34 A1/A2	PCR-RFLP	67.07 ± 9.0	64.07 ± 7.3	YES	HB	6
8.	[31]	India	Asian	170	170	CYP17	34 A1/A2	PCR	67.22 ± 69.31	64.87 ± 68.68	YES	HB	5
9.	[32]	Italy	Caucasian	588	580	CYP17	34 A1/A2	PCR-RFLP	69 ± 6	66 ±7	YES	HB	7
10.	[33]	India	Asian	50	50	CYP17	34 A1/A2	PCR-RFLP	62.02 ± 9.66	69.20 ± 5.40	YES	HB	5
11.	[34]	China	Asia	452	501	CYP17	34 A1/A2	PCR-RFLP	58.02 ± 7.38	57.28 ± 7.61	YES	HB	7
Studies included for the VDR gene polymorphism													
1.	[15]	India	Asian	160	160	VDR	Taq-I,Bsm-I,Fok-I	PCR-RFLP	64.4 ± 9.1	62.9 ± 9.7	YES	HB	6
2.	[16]	Lebanon	Caucasian	68	79	VDR	Taq-I,Bsm-I, Apa-I, Fok-I	PCR-RFLP	65.98 ± 9.97	58.33 ± 10.14	YES	Mixed	7
3.	[17]	China	Asian	200	200	VDR	Fok-I	PCR-RFLP	73.9 ± 2.7	73.56 ± 2.68	YES	HB	5
4.	[34]	China	Asia	452	501	VDR	Apa-I, Fok-I	PCR-RFLP	58.02 ± 7.38	57.28 ± 7.61	YES	HB	7
5.	[35]	Netherland	Caucasian	93	56	VDR	Taq-I	PCR-RFLP	66.4 ± 7.3	64.4 ± 10.6	YES	HB	6
6.	[36]	Japan	Asian	209	128	VDR	Taq-I,Bsm-I, Apa-I	PCR-RFLP	70.4 ± 9.4	73.5 ± 7.1	YES	PB	6
7.	[37]	Japan	Asian	83	90	VDR	Taq-I	PCR-RFLP	71.8 ± 6.0	67.8 ± 7.2	YES	HB	7
8.	[38]	Thailand	Asian	44	30	VDR	Taq-I,Bsm-I, Apa-I	PCR	NA	NA	YES	HB	5
9.	[39]	China	Asian	189	502	VDR	Fok-I	PCR-RFLP	68.6 ± 68.5	71.7 ± 68.7	YES	HB	6
10.	[40]	Brazil	Caucasian	41	169	VDR	Taq-I,Bsm-I, Apa-I, Fok-I	PCR-RFLP	66.2 ± 10.6	23 ± 4.5	YES	HB	8
Studies included for the ACE gene polymorphism													
1.	[41]	Lebanon	Caucasian	69	69	ACE	I/D polymorphisms	PCR	69.1 ± 8.4	55.8 ± 11	YES	HB	7
2.	[42]	India	Asian	200	200	ACE	I/D polymorphisms	PCR	58.3 ± 6	57.6 ± 3.4	YES	HB	5
3.	[43]	Iraq	Caucasian	75	81	ACE	I/D polymorphisms	PCR-RFLP	56.4 ± 3.9	49.2 ± 6.1	YES	HB	5
4.	[44]	Mexico	Caucasian	20	38	ACE	I/D polymorphisms	PCR-RFLP	NA	NA	Yes	HB	5

### Association between CYP17 (rs743572) gene polymorphism and BPH susceptibility

A non-significant relationship between CYP17 (rs743572) gene polymorphism and risk of BPH was observed under dominant model (*OR* = 0.96, 95% *CI* = 0.87–1.06) and recessive model (*OR* = 0.82, 95% *CI* = 0.60–1.13), respectively (Table 2, Supplementary Fig. S1A). Upon conducting the subgroup analysis based on the ethnicity of the study population, no significant association was observed based on the Asian population under the dominant model (*OR* = 0.96, 95% *CI* = 0.83–1.11) and recessive model (*OR* = 0.77, 95% *CI* = 0.43–1.36); also, on the Caucasian population, no significant association was observed under dominant (*OR* = 0.96, 95% *CI* = 0.83–1.12) as well as recessive model (*OR* = 0.93, 95% *CI* = 0.74–1.17), respectively (Table 2). Overall, no evidence of heterogeneity was observed (*I*^2^ = 0.00%, *P* = 0.67).

**Table 2:** Population-wise and subgroup analyses using different genetic models for CYP17, ACE, and VDR polymorphisms

Gene polymorphisms	Population	Dominant model			Recessive model		
		*OR* (95% *CI*)	*I*^2^	*P* value	*OR* (95% *CI*)	*I*^2^	*P* value
CYP17 (rs743572)	Overall	0.96 (0.87–1.06)	0%	0.07	0.82 (0.60–1.13)	60.2%	0.005
	Asian	0.96 (0.83–1.11)	8%	0.36	0.72 (0.43–1.33)	76.5%	0.0001
	Caucasian	0.96 (0.83–1.12)	0%	0.72	0.93 (0.74–1.17)	0%	0.4
ACE (I/D)	Overall	1.00 (0.75–1.35)	28.5%	0.24	1.67 (1.03–2.73)	26.2%	0.25
	Asian	0.98 (0.76–1.26)	0%	0.72	1.42 (0.99–2.03)	0%	0.53
	Caucasian	1.56 (0.38–6.40)	75.3%	0.04	6.18 (1.38–27.68)	0%	0.73
VDR (fok1)	Overall	0.90 (0.78–1.04)	5.8%	0.37	0.71 (0.54–1.07)	27.7%	0.22
	Asian	0.91 (0.76–1.10)	36.7%	0.19	0.69 (0.47–1.03)	40.8%	0.16
	Caucasian	0.83 (0.56–1.24)	0%	0.52	1.21 (0.50–2.95)	0%	0.35
VDR (bsm1)	Overall	1.05 (0.80–1.39)	35.6%	0.18	1.34 (0.90–1.99)	0%	0.85
	Asian	1.18 (0.73–1.89)	60.8%	0.07	1.29 (0.79–2.19)	0%	0.59
	Caucasian	0.91 (0.63–1.29)	0%	0.78	1.40 (0.77–2.53)	0%	0.53
VDR (apaI)	Overall	1.07 (0.77–1.51)	64.9%	0.02	1.23 (0.57–2.64)	77.2%	0.002
	Asian	1.17 (0.73–1.89)	76.3%	0.001	1.62 (0.56–4.65)	83.5%	0.002
	Caucasian	0.92 (0.50–1.67)	56%	0.13	0.80 (0.40–1.60)	0%	0.43
VDR (TaqI)	Overall	1.16 (0.95–1.42)	8%	0.41	1.36 (0.91–2.02)	0%	0.6
	Asian	1.45 (0.86–2.45)	39.5%	0.19	2.34 (0.74–7.33)	0%	0.85
	Caucasian	0.98 (0.72–1.32)	0%	0.82	0.92(0.52–1.62)	0%	0.74

*Abbreviations*: *OR* Odds ratio, *CI* Confidence interval, *CYP* Cytochrome P450c17alpha, *ACE* Angiotensin-converting enzyme, *VDR* Vitamin D receptor

### Association between ACE I/D gene polymorphisms and risk of BPH

**Fig. 2:**
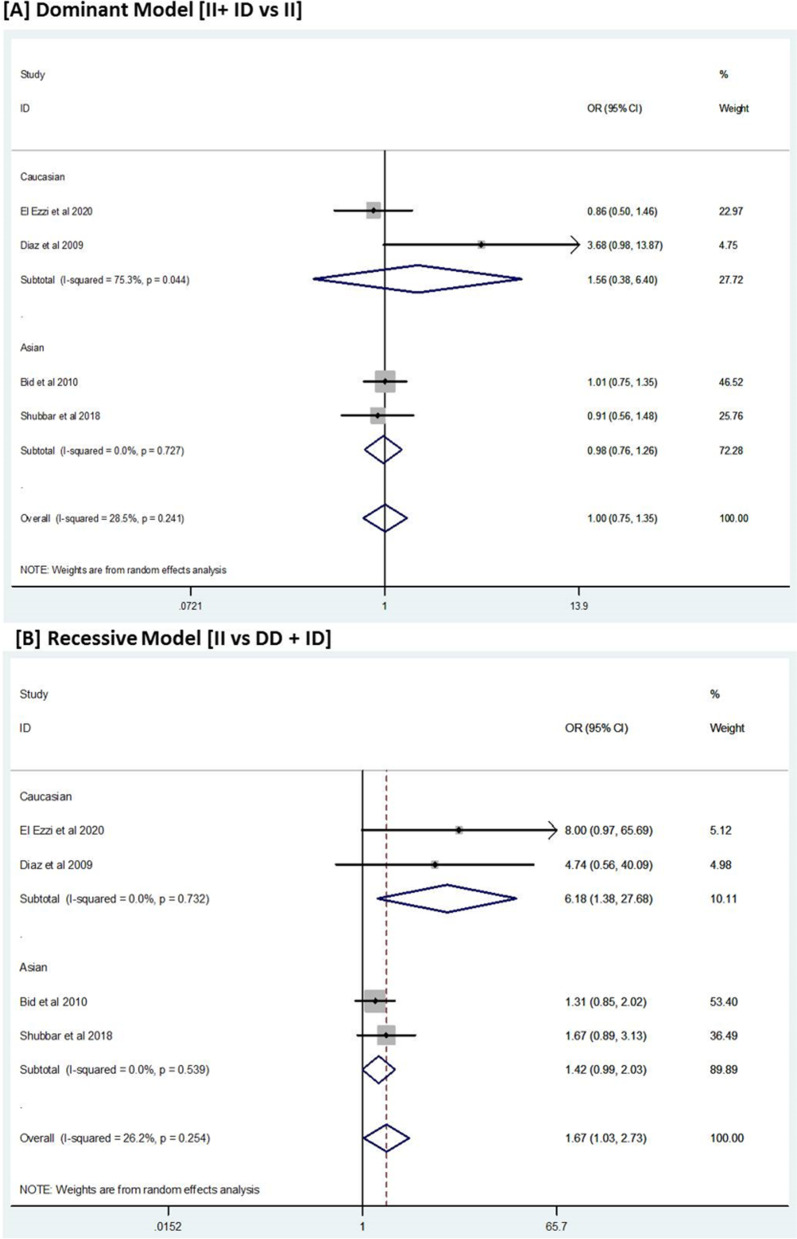
Forest plot of ACE I/D polymorphism and BPH risk. **A** Dominant and **B** recessive models

A significant association between angiotensin-converting enzyme (ACE) insertion/deletion (I/D) polymorphism and risk of BPH was observed under the recessive (II vs. ID + DD) model (*OR* = 1.67, 95% *CI* = 1.03–2.73), but not in the dominant (II + ID vs. DD) model (*OR* = 1.0, 95% *CI* = 0.75–1.35) (Table 2, Fig. 2). Upon conducting the subgroup analysis based on the ethnicity of the study population, a significant association was also observed based in the Asian population under the recessive model (*OR* = 1.42, 95% *CI* = 0.99–2.03) as well as the Caucasian population (*OR* = 6.18, 95% *CI* = 1.38–27.68), respectively. However, no significant association was observed under the dominant model (*OR* = 0.98, 95% *CI* = 0.76–1.35) in Asian as well as in Caucasian (*OR* = 1.56, 95% *CI* = 0.38–6.40) (Table 2, Fig. 2). Overall, slight non-significant evidence of heterogeneity was observed (*I*^2^ = 28.5%, *P* = 0.24).

### Association between vitamin D receptor (VDR) gene polymorphisms and risk of BPH

Four polymorphisms (Taq-I, Bsm-I, Apa-I, and Fok-I) of the VDR gene with risk of BPH from seven case–control studies were identified. Seven case–control studies focused on Taq-I, six case–control studies focused on Fok-I, and five case–control studies focused on Bsm-I and Apa-I gene polymorphisms of VDR. The pooled analyses indicated that these four polymorphisms might not be associated with the risk of BPH.

All four VDR polymorphisms were not associated with the risk of BPH under the dominant model: Taq-I: *OR* 1.16, 95% *CI* (0.95–1.42) for TT + Tt vs. tt; Bsm-I: *OR* 1.05, 95% *CI* (0.80–1.39) for BB + Bb vs. bb; Apa-I: *OR* 1.07, 95% *CI* (0.77–1.51) for AA +Aa vs. aa; Fok-I: *OR* 0.90, 95% *CI* (0.78–1.04) for FF + Ff vs. ff and recessive model (Taq-I: *OR* 1.36, 95% *CI* (0.91–2.02) for tt vs. TT; Bsm-I: *OR* 1.34, 95% *CI* (0.90–1.99) for bb vs. BB; Apa-I: *OR* 1.23, 95% *CI* (0.57–2.64) for aa vs. AA; Fok-I: *OR* 0.76, 95% *CI* (0.54–1.07) for ff vs. FF) (Table 2, Supplementary Figs. S3A–S6A). Subgroup analysis according to ethnicity also did not confirm any risk of association for all these four polymorphisms and the risk of BPH in both Asian as well as Caucasian populations. No heterogeneity for all these four polymorphisms was observed.

### Publication bias

Begg’s funnel plot and the Egger test were performed to assess the publication bias arising from the literature for all three genes under study. No obvious asymmetry was observed in any genetic model in any of the genes according to the visual assessment of the funnel plot (Supplementary Figs. S1B, S2B, S3B–S6B). In addition, there was no statistical evidence of publication bias among studies using Egger’s regression test.

### Sensitivity analyses

Furthermore, we performed sensitivity analyses to assess the influence of each individual study of every gene on the pooled ORs by sequential omission of individual included studies. However, the corresponding pooled ORs were not significantly altered by removing any individual study except for allelic models of all three genes (Supplementary Figs. S1C, S2C, S3C–S6C). Therefore, the sensitivity analysis confirmed that the results of this meta-analysis were statistically reliable and robust.

### Meta-regression analysis

Meta-regression analysis based on the quality scores for the relationship between all three gene polymorphisms and the risk of BPH did not confirm any deviation of the findings (Supplementary Fig. S1D).

## Discussion

BPH affects the quality of life of patients including both young to old men. Its aetiology has been described by multiple hypotheses of the involvement of several genetic and metabolic factors. Polymorphism in several genes has been linked to the high susceptibility of BPH. For example, SNPs in CYP17, CYP19, VDR, and SRD5A2 [34] and in chemokine genes CCR2 (rs1799864) and CCL5 (rs2107538) [45] genes have been reported in BPH. Individuals with metabolic syndrome (MetS) or its individual components—including central obesity, hyperinsulinemia, insulin resistance, and dyslipidemia, are more prone to develop BPH and LUTS [46–48]. However, the molecular and stromal mechanisms involved in the pathogenesis of BPH have not yet been fully elucidated. Genetic polymorphisms in vital genes of metabolic pathways associated with BPH impact the phenotype and its severity. Therefore, in the present study, we systematically reviewed and analyzed genetic variations in important genes towards the susceptibility of BPH.

Our systematic review and meta-analysis evaluated the significance of three highly frequent polymorphisms and their association with the risk of BPH by pooling 11 studies for CYP17 (cases = 2078, controls = 2110), 10 studies for VDR (cases = 1539, controls = 1915), and 4 studies for ACE (cases = 364, controls = 388) across two different ethnicities (Asians and Caucasian) (Table 1). The findings from our analysis reveal that genetic polymorphism in the ACE gene was significantly associated with the risk of BPH when compared with control subjects, whereas the polymorphism located in VDR and CYP17 genes failed to do so (Table 2, Fig. 2).

The hydroxylase enzyme encoded by the CYP17 gene regulates steroid hormone synthesis and may play a crucial role in the etiology of hormone-related cancers such as prostate cancer and breast cancer. There is no consensus on the effect of genetic polymorphisms of these genes on BPH susceptibility. For example, the A1 allele with a gene dosage effect and -34T>C polymorphisms of CYP17 have been associated with an increased risk of BPH and its clinical progression, while no positive association was found in Orientals [49]. Similarly, the VDR gene polymorphism was not found significantly associated with BPH in Asians and Caucasians [18], whereas a significant association was demonstrated by two variants (Taq-I and Bsm-I) in Asians [15] and the other two variants (ApaI and BsmI) in Lebanese men [16]. However, by pooling these and similar shortlisted reports, in the present analysis, no significant association was observed with the BPH susceptibility. Even after omitting one of these studies (either CYP17 or VDR), we observed that the overall effect size did not change significantly in the leave-one-out analysis ordered by both heterogeneity and effect size. Similar results were observed in the analysis of VDR polymorphism.

However, in the case of analysis of ACE gene polymorphism for BPH susceptibility, we found a significant association between them. The ACE gene is involved in the hyperactivity of local RAAS in the prostate and has to be demonstrated to be involved in the pathogenesis of BPH. The insertion/deletion polymorphism of the ACE gene is directly related to ACE plasma levels [50]. This variation has also previously been found to be associated with LUTS or surgery for LUTS in study populations of Mexico and India [43, 45]. Thus, such reports prove the robustness of the current study.

Publication bias and heterogeneity could in turn distort the results of the meta-analysis. However, the publication bias was not detected in Begg’s funnel plots for per-allele models or their combinations for all three genes. In addition to bias, heterogeneity was also not found in the current study, which could in turn distort the results of the meta-analysis. These all further strengthen our results.

## Conclusion

We found a significant association of ACE gene and negative association of CYP17 and VDR gene polymorphisms with the risk of BPH, which patients with ACE polymorphism (recessive) are more susceptible to BPH onset. Although relatively few studies on ACE polymorphism than VDR/CYP17 genes were analysed in the present study, large studies with prospective data and large sample size should be conducted.

## Supplementary Information

**Additional file 1. Figure S1.** Meta-analysis of included studies reporting on CYP17 polymorphism in BPH susceptibility compared with controls. **Figure S2.** Meta-analysis of included studies reporting on ACE gene polymorphism in BPH susceptibility compared with controls. **Figure S3.** Meta-analysis of included studies reporting on VDR Taq1 polymorphism in BPH susceptibility compared with controls. **Figure S4.** Meta-analysis of included studies reporting on VDR bsm1 polymorphism in BPH susceptibility compared with controls. **Figure S5.** Meta-analysis of included studies reporting on VDR APa1 polymorphism in BPH susceptibility compared with controls. **Figure S6.** Meta-analysis of included studies reporting on VDR Fok1 polymorphism in BPH susceptibility compared with controls.

## Data Availability

The datasets generated and/or analyzed during the current study are available from the corresponding author on reasonable request.
