# Supplementary material for: Systematic review and meta-analysis of candidate gene association studies of benign prostate hyperplasia
Source: Syst Rev. 2022 Apr 5;11:60. doi: 10.1186/s13643-022-01914-7 (PMC8985373; doi:10.1186/s13643-022-01914-7)
Supplement: Supplementary file 1 — Additional file 1. Figure S1. Meta-analysis of included studies reporting on CYP17 polymorphism in BPH susceptibility compared with controls. Figure S2. Meta-analysis of included studies reporting on ACE gene polymorphism in BPH susceptibility compared with controls. Figure S3. Meta-analysis of included studies reporting on VDR Taq1 polymorphism in BPH susceptibility compared with controls. Figure S4. Meta-analysis of included studies reporting on VDR bsm1 polymorphism in BPH susceptibility compared with controls. Figure S5. Meta-analysis of included studies reporting on VDR APa1 polymorphism in BPH susceptibility compared with controls. Figure S6. Meta-analysis of included studies reporting on VDR Fok1 polymorphism in BPH susceptibility compared with controls. [file 13643_2022_1914_MOESM1_ESM.docx]

**Supplementary Figures**

**Figure-S1**: Meta-analysis of included studies reporting on CYP17 polymorphism in BPH susceptibility compared with controls.

**[A] Forest plot**

**(i) Dominant Model [A2A2 + A1A2 vs A1A1]**

**(ii) Recessive Model [A2A2 vs A1A1 + A1A2]**

**[B] Begg’s Funnel Plot**

**[C] Sensitivity Analysis plot**

**[D] Meta regression plot based on NOS quality Score**

 **Figure S2:** Meta-analysis of included studies reporting on ACE gene polymorphism in BPH susceptibility compared with controls.

**[A] Begg’s Funnel Plot**

**[B] Sensitivity Analysis plot**

**Figure- S3:** Meta-analysis of included studies reporting on VDR Taq1 polymorphism in BPH susceptibility compared with controls.

**[A] Forest plot**

**(i) Dominant Model**

**(ii) Recessive Model**

**[B] Begg’s Funnel Plot**

**[C] Sensitivity Analysis plot**

**Figure-S4:** Meta-analysis of included studies reporting on VDR bsm1 polymorphism in BPH susceptibility compared with controls.

**(A) Forrest plot**

**(i)Dominant Model**

**(ii)Recessive Model**

**[B] Begg’s Funnel Plot**

**[C] Sensitivity Analysis plot**

**Figure- S5:** Meta-analysis of included studies reporting on VDR APa1 polymorphism in BPH susceptibility compared with controls.

**[A]Forest plot**

**(i ) Dominant Model**

**(ii) Recessive Model**

**[B] Begg’s Funnel Plot**

**[C] Sensitivity Analysis plot**

**Figure-S6**: Meta-analysis of included studies reporting on VDR Fok1 polymorphism in BPH susceptibility compared with controls.

**[A]Forest plot**

**(i) Dominant Model**

**(ii) Recessive Model**

**[B] Begg’s Funnel**

**[C] Sensitivity Analysis plot**
